# Supplementary material for: Trend and determinants of quality of family planning counseling in Ethiopia: Evidence from repeated PMA cross-sectional surveys, (2014–2019)
Source: PLoS One. 2022 May 27;17(5):e0267944. doi: 10.1371/journal.pone.0267944 (PMC9140310; doi:10.1371/journal.pone.0267944)
Supplement: S1 Appendix — (PDF) [file pone.0267944.s001.pdf]

Appendix

Table (A1). Percentage (weighted) of women who received family planning counseling by selected covariates (1, 2, and 3 represents women who got one, two and three counseling services†, respectively.)

Factor	2014(n=3886)			2015(n=2450)			2016(n=2503)			2017(n=2366)			2018(n=2353)			2019(n=2036)		
	1	2	3	1	2	3	1	2	3	1	2	3	1	2	3	1	2	3
Total	24.4	10.9	27.6	21.1	8.1	38.6	24.0	11.4	32.2	23.8	8.9	31.6	23.0	11.2	29.0	32.3	10.7	11.8
**Residence**																		
Urban	26.1	9.1	29.7	23.0	8.8	37.4	22.4	9.9	34.1	24.1	8.2	29.5	24.0	8.1	27.0	31.0	10.4	10.8
rural	23.8	11.6	26.8	20.4	7.9	39.1	24.6	12.0	31.5	23.6	9.2	32.3	22.0	12.4	30.0	34.8	11.1	13.7
**Region**																		
Tigray	29.4	9.3	35.1	30.9	10.0	34.9	34.5	11.7	32.4	25.0	12.4	34.3	27.0	9.3	35.0	35.5	11.6	14.6
Amhara	26.9	11.2	16.6	23.5	10.0	28.8	27.6	9.6	19.8	27.8	5.1	28.6	25.0	9.4	21.0	32.9	10.7	6.2
Oromia	25.5	12.4	26.4	22.7	7.5	39.3	25.2	13.4	30.6	20.4	10.9	27.7	25.0	11.5	27.0	34.4	11.5	11.7
SNNP	18.2	10	39.5	14.7	6.5	49.3	17.4	11.3	49.2	23.6	11.1	40.7	16.0	14.6	39.0	28.0	8.9	13.9
Addis Ababa	22.8	8.7	41.4	23.9	7.7	33.5	17.7	10.0	36.9	21.3	9.3	38.9	18.0	12.3	41.0	29.8	10.7	23.3
Other*	22.1	7.8	28.8	12.0	5.5	51.0	22.3	12.9	26.7	20.6	8.2	23.7	19.0	6.1	36.0	27.4	10.4	23.5
**Wealth Quntile**																		
Lowest	21.7	10.7	28.2	18.9	8.7	36.8	23.6	12.5	33.6	22.5	9.8	32.5	19.0	15.4	29.0	30.0	10.1	11.8
Lower	21.5	11.7	26.6	19.7	7.8	34.4	23.5	10.4	26.7	21.6	8.3	34.7	22.0	11.9	28.0	34.3	8.2	7.4
Middle	26.4	11.1	24.9	20.5	8.5	41.2	26.2	12.7	28.8	21.4	7.9	25.4	22.0	13.0	31.0	28.9	12.0	12.1
Higher	25.8	10.8	26.0	19.8	8.4	41.1	25.0	11.6	35.5	27.9	11.0	30.8	28.0	9.7	26.0	32.3	10.6	11.1
Highest	24.9	10.6	30.8	24.7	7.6	38.7	22.3	10.3	34.9	23.7	7.6	33.2	22.0	8.3	31.0	35.2	11.7	15.3
**Education**																		
Never attained	24.0	12.5	25.0	20.9	6.0	39.2	24.7	12.0	30.3	21.3	9.2	33.4	24.0	11.9	28.0	30.4	10.4	10.8
Primary	24.6	8.9	29.0	22.9	11.0	35.1	24.8	10.6	31.9	25.0	9.7	29.3	22.0	11.3	30.0	31.5	9.1	11.2
Secondary/higher	25.3	10.7	32.0	18.2	8.3	43.7	20.7	11.7	37.0	26.3	7.0	32.1	24	9.6	29.0	36.7	13.9	14.3
**Age (year)**																		
15-19	21.3	10.2	23.9	16.7	8.1	29.8	20.6	8.3	20.2	24.3	6.4	15.6	26.0	9.5	17.0	32.0	10.5	6.1
20-24	26.3	11.7	23.2	19.6	9.5	35.7	28.6	10.5	25.6	22.0	10.8	27.4	21.0	9.8	25.0	34.3	8.5	9.7
25-34	25.3	9.6	28.2	23.0	7.2	40.1	22.5	11.0	36.2	26.6	8.8	33.2	24.9	12.0	31.4	30.6	11.9	14.3
35-49	22.3	12.9	30.9	20.8	8.5	41.3	24.2	13.6	33.9	20.0	8.6	38.1	20.1	11.5	31.5	33.8	10.4	11.1
**Marital Status**																		
Never married	22.7	12.3	16.7	18.4	12.0	25.8	27.7	7.6	18.4	12.2	3.6	19.6	17.0	4.5	17.0	22.9	5.9	10.8
Married/in union	23.9	11.0	28.4	21.1	8.1	39.9	23.7	11.3	33.4	24.1	9.3	32.6	23.0	11.4	30.0	33.2	11.2	11.6
Divorced/widowed	33.7	9.1	22.3	24.0	5.2	30.4	26.2	15.4	25.3	27.0	6.8	24.9	19.0	13.3	24.0	20.9	4.2	15.8
**Parity**																		
0 Child	24.2	9.2	19.9	19.4	8.4	29.0	21.5	7.8	22.0	20.6	5.5	17.0	18.0	9.1	18.0	25.5	5.8	8.9
1-2 children	25.2	10.1	28.7	20.5	8.0	39.8	25.1	11.4	32.1	27.1	9.5	30.8	26.0	10.1	29.0	34.7	10.8	12.9
3+ children	24.0	11.8	28.5	22.0	8.2	40.6	23.8	12.2	34.5	22.1	9.5	36.9	22.0	12.7	33.0	31.6	11.7	11.3
**Method source**																		
Hospital	24.2	9.9	20	22.2	8.6	26.4	15.8	11.4	29.8	24.3	6.7	19.3	20.7	10	19.1	37.6	13	16.3
Health post	21	13	28.8	18.2	6.9	48.7	25.7	12	35.1	22.9	10.6	37.2	26	11.4	28.9	34.9	10.4	10.5
Health center	27.6	10	31.3	21.9	9.6	40.3	25.8	12.7	36.2	24.1	9.6	37	23.4	13.4	37	32.1	11.4	12.8
Pharmacy	21.9	6.5	10.3	17.6	5	13.5	11	6.5	11.5	15.5	3.8	11.6	10.2	2.6	6	13.1	1.3	0.7
Others	18.7	17.5	24.8	23.4	9	36.3	18.8	8.2	21.5	18.4	9.6	23.6	23.4	5.4	25.2	34.9	5.6	10.7
**Method type**																		
Short-acting	25.0	10.4	24.6	22.4	7.4	35.5	24.5	11.7	26.0	24.9	7.6	27.0	24.0	8.2	26.0	33.7	9.6	9.6
Long-acting	24.4	12.1	40.6	21.1	12.0	48.0	20.9	12.8	47.8	18.1	12.3	47.2	19	18.2	40.0	29.8	12.6	15.7
**Media Exposure**																		
No	25.7	11.1	20.7	22.5	7.5	32.8	25.3	10.8	25.3	26.0	8.8	27.3	25.0	10.3	24.0	30.7	8.7	9.1
Yes	23.0	10.6	35.2	19.7	8.9	45	22.8	11.8	40.0	21.1	8.9	37.3	21.0	12.5	36.0	34.6	13.6	15.6

†The three questions used to measure couselling service are: (a) told about other methods, (b) counseled on side effects, and (c) counseled on what to do if side effects were to occur. * **Other**- region represents the region Afar, Somali, B-Gumuz, Gambella, Harari, and Dire Dawa.
