# Supplementary material for: Trend and determinants of quality of family planning counseling in Ethiopia: Evidence from repeated PMA cross-sectional surveys, (2014–2019)
Source: PLoS One. 2022 May 27;17(5):e0267944. doi: 10.1371/journal.pone.0267944 (PMC9140310; doi:10.1371/journal.pone.0267944)
Supplement: Attachment — Submitted filename: Response to Reviewers.pdf [file pone.0267944.s003.pdf]

Response to Editor and Reviewers

Editor Comments

Family planning is such a very important service. Here the authors report a longitudinal analysis of quality counselling using PMA surveys. I applaud the authors for first, use longitudinally a series of 7 cross-sectional datasets, secondly for defining just one ordinal outcome rather than making 3 logistic regressions (as many would do). This is a very efficient use of data. However, it is still debatable to use just these 3 variables as indicators of the quality of counselling. I see no discussion of this.

Issues

I. Abstract:

Please split this into introduction, methods, results and conclusions

The abstract section updated accordingly.

II. Introduction

This is a very good background. Just a clarification on line 12 what are these numbers (0.77 to 3.17)? Are these yearly changes?

- It is an annual rate of changes (percentage points) in modern contraceptive prevalence rates among all women of reproductive age (15–49 years).

III. Methods

1. In the “study design and data source” we are pointed to reference 26 to learn the sampling details of PMA2020. That is fine but brief details of such sampling should be added here. It would be great if details of what is the size of an Enumeration Area or of the PSU relative to an administrative area in Ethiopia. How is comparable?

- In the revised version of the manuscript, additional clarification in this issue was given.

2. What is this method of information index? (lines 77/78)

- It is a composite score for family planning service quality.

3. Please add spaces at “if a woman got allthethree” between lines 84 and 85

- Thanks for pointing this, updated accordingly.

4. Equation 2 - nowhere is explained the meaning of sigma2 squared (the variance of the random-effects).

- The variance of the random effect (\sigma_{2}^{2}) represents how much variability there is between clusters with respect to the response variable.

5. Table 1 - please either specify what “svy-year” means or write “survey year”.

Updated.

6. Line 122 - the Brant is a name. So please correct it to be “Brant test”.

- Corrected.

7. Line 136 - Stata is not an acronym. So correct STATA to Stata please. See the official documentation for reference. For example, your reference 34 clarifies that.
   - Corrected.

8. Line 137, I suggest adding the reference for ggplot2. Your plots are based on ggplot2. Correct?
Yes, and reference on ggplot2 included.

9. Was any goodness of fitness performed in this analysis?
- Yes. Likelihood ratio test was employed to assess the importance of incorporating the random effect term in the model. Further, AIC was used to compare models with different covariates.

IV. Results

1. What is the amount of missing data here?
- Our analytical samples are all women who completed the survey questionnaires. counseling service.

2. Table 2 please on the caption/footnote that these are proportions in percentages.
- Are these weighted proportions? Yes
- all percentages seem to respect the 2 decimal places. So why not “2014 FP message” “2015 FP Facility”? Corrected.

3. Table 3 there are empty cells. Such as 2019 other regions. Can you check this, please?
   - Checked and addressed.

4. Line 166 no figure or table indicated.
   - Addressed.

5. How the confidence intervals for the relative changes are computed?

   - Using the linearization approach.

6. Table 5 - What are COR and AOR? Put in the footnote, please. Here should be the place to add the variance component proportion. In fact, I do not see this in the results.

- COR=Crude Odds Ratio, AOR= Adjusted Odds Ratio

V. Discussion:

Why no limitations discussion here?

- In the revised version of the manuscript, a limitation section included.

Reviewer #1:

The author chose a very important topic and I admire her/his statistical knowledge and skills. I would very much like to see a paper emerge on this subject. However, I feel that the authors have missed the mark in several respects that I outline below. I recommend that the authors take my feedback into consideration and determine how best to proceed themselves. The description of the data was so inadequate that I would not have been able to make my way through the paper had I not been somewhat familiar with the data source myself; however, my feedback might have missed the mark also since I had to make assumptions about the data source. Still, I would not reject a paper on this topic with these data, taking into account what else I know about PMA 2020 data but I think the analysis presented here with cannot go forward without first presenting the data source and collection methods more clearly, more thoroughly conceptualizing the notion of FP counseling quality in the wider context of FP service quality and mapping the factors that influence it vis-à-vis the array of indicators in the data sources available, and then thinking through more soundly what factors are most feasible and relevant for their analysis given the data at hand given the possibility of biases.

Abstract

- I would not refer to 2014 as "baseline" and 2019 as "endline" since that implies that there was an intervention in between the years.
- "A multi-level model was used to examine potential factors". I would state your research question(s) related to exploring trends and associations more clearly and for each the corresponding statistical strategy you use to answer the question.
- What is the operational definition of "good family planning counseling" and "poor family planning counseling"? How did you construct it as a variable in the statistical analyses?
- For readers that do not know indicate briefly the type of data that you are using (i.e., household surveys, facility-based exit interviews).

- In the revised version of the manuscript, the abstract entirely updated based on the Editor and reviewer comments.

Background

"In sub-Saharan Africa countries the trend in using 10 modern contraceptive method among women of reproductive age varied substantially from 0.77 to 3.17" – I do not understand this sentence. What is meant by 0.77 to 3.17?

- It is an annual rate of changes (percentage points) in modern contraceptive prevalence rates among all women of reproductive age (15–49 years).

"Prevalence of family planning coverage" – What is this? Prevalence to me connotes the level of method use among individuals in a population and coverage implies that amount of population that has access to a service (two different things). Similarly in line 15 you say "coverage" of contraception is associated with individual-level covariates. Do you mean "use of contraception"?

- Yes, to mean "use of contraception", and updated accordingly.

The author frames the background with the global situation of contraception prevalence and unmet need in broad terms and leaps to a discussion of very focused set of factors that influence method use, counseling. A more useful framing might start by quickly acknowledging gaps in availability and use of FP services among women in sub-Saharan Africa, and proceed to frame the paper around the need to better understand the quality of care – pointing out the multiple aspects of family planning service quality and a overview of the approaches that have been used to measure and evaluate FP service quality, evidence on the determinants and consequences of FP service quality, then introducing the subject of this paper – the determinants of FP counseling quality - in terms of the particular knowledge gap that it is filling.

- We greatly appreciate the feedbacks, and the background section updated.

Be sure to clearly define family planning counseling and how the quality of FP counseling has been defined and studied in the literature. There are multiple dimensions to it and ways of measuring it (depending on whether it is explored from the client or provider perspective, whether the focus is on technical information imparted during the exchange, the rapport between client and provider, the service delivery environment and its privacy and availability of other information such as leaflets, client recall of specific messages about side effects versus their general recall as to whether more than one method was discussed, use of mystery clients, direct observations, client-exit interviews immediately after the service, household surveys that occur much after the service exchange, assessments of provider biases and knowledge, etc.). It is crucial to acknowledge this and describe this paper's definition of FP counseling quality in that context. The current draft seems to assume that FP counseling quality and information exchange are the same thing when in fact, I think, the latter is an aspect of the former.

Then proceed to explain the choice to focus specifically on the "information exchange", why this is novel and appropriate. Some useful information for this is already in the paper, but it is not clear what dimension of quality and family planning quality that they measured and report on.

"Studies on family planning counseling service in Ethiopia showed that 40 the coverage is below 40%" – What does this mean exactly?

- Percentage of women who received high quality family planning counseling service is below 40%.   The sentence updated in the revised version of the manuscript.

Lines 42-49, make clear the type of data you are talking about when you are citing the literature. Were these household surveys in which women were asked to recall information about the last time they sought FP services? Or were they exit-interviews conducted immediately after women were discharged from care? Did the surveys assess quality from the client or provider perspective (i.e., who did they enroll)?

- It is a household surveys in which women were asked to recall information about the last time they sought family planning counseling service. So, the survey assesses the quality from client perspective.

Materials and Methods

“The PMA surveys are repeated cross-sectional surveys based on a multistage stratified cluster sampling design.” Who do they interview and where? What is the operational definition of the enumeration area” and what is the operational definition of “population proportion to size” for these surveys? What does the term “multistage stratified cluster sampling design” mean for this study specifically (define the stages, define the cluster and strata). Did the sampling frame ever change during the seven years of survey implementation and if so how often? I prefer to see this information in the paper and not refer to another paper. I am familiar enough with PMA 2020 to know that it is well done, but I find this information rather crucial to understand the rest of the manuscript so I think you should provide it.

- Thanks for the feedback, and updated accordingly.

You refer to “the outcome variable” in line 70 but at this juncture I do not think that you have formally defined it yet.

- Addressed.

Variables: I find this section extremely difficult to understand because I do not know if the survey respondents were women of reproductive age enrolled in households, actual clients enrolled immediately after discharge from the service, providers that delivered the service and report of their own performance. Since I am familiar with PMA 2020 I assume you mean WRA enrolled at the household, so I can make it through the manuscript. But describe the sample clearly and how they are distinguished from the underlying population. Presumably your sample is a sub-sample of women from the surveys that had received FP services within some time frame before the survey, or are they simply reporting on the last time they ever sought FP services? Are they all FP users or acceptors or just anyone that received any counseling? Clarify the range of the duration representing the time between when they index counseling interaction took place and the time of the interview.

- Thanks for this comment. In the revised manuscript, study samples were clearly described.

Table 1: The third age category seems quite broad. I suggest breaking it down to 25-34, and 35+. What about permanent methods. Are you able to find out if they are new or return method users? Media exposure: do you mean exposure to media before or after the service episode. This may be a minor point, but consider whether it is relevant that the client-level factors represent the status of the client at the time of the interview and not when they obtained family planning.

- We categorized age as adolescent, young and adult for interpretation purpose. In the revised version we consider your comments, and recategorized age as: 15-19, 20-24, 25-34 and 35-49.
- Media exposure before the service episode.

I encourage the author to map out a causal pathway model between each proposed “exposure” and the “outcome” (quality of information exchange) taking into consideration the possibility of the exposure occurring after the dependent variable is mentioned, the theoretical linkages between the exposures and the quality of FP counseling, and possible recall bias. Its not necessary to include this in the paper, but the author may find it beneficial to do so and it is up to her/him.

In this vein, some issues to consider:

- Some of the client level factors may predispose clients to be more or less assertive during the interaction with provider (e.g., ask questions, request clarifications, inquire about different methods they have heard of). In this case, would the outcome reflect variation in the quality of counseling or rather the common sense that information exchange is better when clients ask questions.
- Some of the client level factors predispose some clients to poorer recall of counseling information than others.
- I am concerned about "FP discussion" as a exploratory variable (presumably you mean independent variable, possibly a predictor of interest)? How is an "FP discussion" different from the information exchange whose rating is treated as the outcome? Is this not problematic for your model?
- Thanks for raising this issue. We acknowledge your comment, and in the revised manuscript, we drop this variable from our analysis/model.
- I would include type of facility since I know they are many levels of care in the Ethiopian healthcare system (e.g., health post, health center, hospital, pharmacy, community based distributor).
- Thanks again. Yes! We did it now.
- Also it makes a difference if the woman is receiving a re-injection or refilling a prescription of oral contraceptives (she may not need counseling since she has been on the method for some time anyway, and I imagine you'll find that counseling quality is lower for short-term methods for that reason).
- Yes, that is true.
- Can you include cadre type in the model (e.g. health extension worker, nurse, physician)?
- The point you raised is interesting, but we don't have this data in this survey.
- The satisfaction variable seems problematic to me for the same reasons that I pointed out with respect to the "FP discussion" – it seems like a dimension of FP counseling quality in the same way that the information exchange is and I do not see how one can sensibly include in both sides of the equation.
- Thanks for raising this issue. We acknowledge your comment, and drop this variable from our analysis/model.
- Some one that received information on contraceptive method from a provider, especially a user of a method, may be more likely to report media exposure that occurred after the service because they were predisposed to being interested in family planning.
- Similarly exposure to media before the service may prompt women to seek family planning services and ask questions during the exchange with the provider. Thus, (1) there may be the problem of reverse causality, and (2) does this really tell us anything about the quality of counseling or rather the effect of media exposure on recall, health behaviors and assertiveness of client during exchanges with provider?
- Having media exposure on family planning may help the client to seek more information on the three key question (MII defined) from the provider which ultimately improve the quality of counseling.

- As you think through the causal pathway model, consider other factors such as whether the facility had commodities and supplies when the client sought FP services, whether the provider had ever received FP training and when.
- Thanks for the feedback. In the future, we will consider to collect such data in the upcoming surveys.

Statistcal analysis

Describe in greater detail how the “data analysis methods considered sampling weights for generating unbiased population estimates” and why the authors believed that this was necessary in the multi-level model with covariates? How did you incorporate the design-based approach to address unequal sampling probabilities in the multilevel model? Reading farther into the manuscript I see that you (might) have gone through the weighted procedures to obtain the descriptive results, but this also needs to be explained more clearly. Make very clear what were the different components of your analysis and the specific methods you implemented for each respectively. Generally, the lack of clarity in this manuscript has made reviewing this article time consuming and difficult. The authors should have their work reviewed by peers that can give this feedback before submitting to journals.

Include a statement that in as complete and plainspoken terms as possible gives the interpretation of the relationship between your independent and dependent variables that your analysis seeks and how the POM model ascertains this. The application of the POM to longitudinal data with repeat cross-sections is novel and interesting, but complex and readers should be guided in understanding how to interpret that aspect of the model as well. But, a reader that has never done ordered logistic regression but is otherwise conversant in regression will not be able to understand what you are saying. If I had not recently done a similar analysis myself I would be very confused.

Did you apply the Brant test to both models or just model 2?

- In both models. But now, since we drop FP discussion from our model, and re-categorize method source as Hospital, health-center, health-post, pharmacy and other, the proportional odds assumption satisfied.
-

Results

Table 3 is dizzying. I suggest you innovate with plots to convey the most important trends through a series of carefully tailored visualization, and elucidate these with parsimonious commentary and include this table as a Appendix only.

- We considered your suggestion and updated accordingly.

I think you need to reconsider the findings in light of some of the comments I made above about recall biases, counseling for STM v. LARC, reverse causation in association between media exposure and the outcome and what this tells us about counseling quality, the appropriateness of “FP discussion” and “satisfaction” as predictors, and absent data on the readiness for FP service

delivery (available methods in stock, trained providers). In my opinion, the findings section needs to be strengthened quite appreciably to withstand that critique.

Your explanation of the model results does not reflect the ordered nature of the response variable. How do you interpret the OR in terms of the predictor response association reported by the POM?

- Thanks for pointing this. Updated.

Discussion

I suspect that this section may change significantly upon reflection of my comments above. The trend in the quality of the information exchange during FP counseling is noteworthy and merits elucidation in a published paper, but it seems that this analysis has missed the mark.

There is no question that provider bias is at play, but is it really fair to point to this as the driver of the national trend of declining FP counseling quality when the analysis does not acknowledge anything about the providers' background, training, working environment, access to commodities and appropriate supervision?

Are LARC available in the private sector and if so is this restricted to particulate social franchises? Or are they mostly available in the public sector. It makes sense that LARC provision is associated with associated with more information provision since they entail removal services (unlike oral and injectable methods) and often times these occur in public sector settings. This does not mean that public sector providers are better counselors but the specialize in services that oblige them to share more information.

- As you suggested above, in the revised version of the manuscript, rather than public Vs private, we categorize the method source by facility type.

The issue of recall bias needs to be addressed squarely acknowledging the possibility that respondents may conflate the information they receive about FP methods from their provider with FP information that they heard from another source either before or after they had received FP services.

- Yes, that is true and we mentioned this fact under the limitation Section of the manuscript.

I think a more interesting analysis would focus on the individual characteristics of the provider (training, age, job title, sex, etc.), working environment (facility type, access to commodities, supervision, etc.), and macro-context (region, etc.). Pinpointing the quality of information that comes from provider to the client on characteristics of the client raises to the fore issues of provider bias that we already know about but cannot disentangle from the possibility that women with certain characteristics are more likely to have better recall than others.

- We agree with your suggestions, but in our survey considered for this analysis, we don't have data about the individual characteristics of the provider linked with each client.
