# Supplementary material for: Trend and determinants of quality of family planning counseling in Ethiopia: Evidence from repeated PMA cross-sectional surveys, (2014–2019)
Source: PLoS One. 2022 May 27;17(5):e0267944. doi: 10.1371/journal.pone.0267944 (PMC9140310; doi:10.1371/journal.pone.0267944)
Supplement: Attachment — Submitted filename: Response to Reviewers.pdf [file pone.0267944.s004.pdf]

Thank you for the opportunity to respond to reviewer comments. We thank the reviewer for a thorough secondary review.

**Additional Editor Comments:**

This is an important analysis as I expressed in the previous iterations. The manuscript has improved and the authors addressed most of my comments/questions.

My previous question remaining or with unsatisfactory response:

1. I did ask about the missingness before. Clarifying, what is the response rate of these PMA surveys? What was done if there were participants with missing data among the variables included for this analysis? This could be placed between the current lines 77 to 83.

Thank you to the reviewer for catching this and we apologize for the oversight in the previous version. We have added the text below on page 3 in the revised clean manuscript.

- In PMA surveys considered in this analysis, the response rate is high across different survey years which ranges from 95.6% to 99.2%. The analysis was done for women who completed the survey. If a woman replied "Don't know or No response", these values were recoded as missing.

**New few issues:**

1. The reviewer below, points out the need for some introduction of the concept family planning (FP) service quality and how that end be measured as just 3 questions.

We have expanded our discussion of the Method Information Index in the manuscript. While we agree that three questions are unlikely to capture the full complexity of the concept of quality, the Method Information Index is a well-established indicator in the field of family planning and generally accepted as the current standard for measuring quality. We have included additional information on the creation and validation of the indicator in the Background section and in the abstract.

"Counseling plays a key role in enhancing family planning services, providing contraception related information and supporting family planning and fertility goals for women of childbearing age [4]. The Method Information Index (MII) is the current standard proposed by the Track 20 initiative to monitor quality of family planning counseling [5, 6]. The simple index uses three questions -whether the respondent received counseling on multiple contraceptive methods, whether she was told about side-effects, and if so, whether she was told what to do about side-effects that are available in the majority of population-based surveys, including the Demographic and Health Survey (the DHS) and Performance Monitoring for Action (PMA) [7]. Adaptations have been proposed to the MII, including a fourth question assessing whether women were told they could switch to another method. This question was added in PMA 2019 Survey, however, and for trend purposes is not included in the current analysis."

2. Line21: What is this coverage? Is this high quality of FP coverage?
   - Thank you. Yes, this has been updated.
3. Line 63: the SNNP abbreviation is used for the first time here. Can you write fully what it means?
   Yes done. SNNP stands for Southern Nations, Nationalities, and Peoples'
4. Just for clarification: was the year of survey included in the model as a categorical variable. Correct? Can you clarify that in table 1.

- Yes, and updated accordingly.

5. Results related to table 2. Can you clarify how the weights of the two surveys were combined for 2014 analysis?

- Since the two surveys in 2014 conducted at the beginning and end of 2014, we recalculate the weights using the projected women in reproductive age (15-49) population in 2013.5 and 2014.5 which is 22326082 and 23198829, respectively. See the details below (under #7) how it is calculated.

6. Lines 159: what were the conclusions of the Brant tests? Was the PO assumption kept across all variables?

- Yes, we have included this on page 6.

7. The paragraph 171 to 174 states that all analysis used the sample weights. This leads to 2 questions:

a. How multiple survey weights were combined for this analysis?

- Following the recommendations by Ruilin Ren who is a senior sampling statistician at ICF, we recalculate the survey weights to compute the percentage of women who got high quality family planning counseling service as follows.

  denormalizedWeight=FQweight×(total females age 15-49 in the country at the time of the survey)/(number of women age 15-49 interviewed in the survey)

- Normalizeweight= denormalizedWeight/sum(denormalizedWeight)

- Where, FQweight represents the computed weight in each survey.

| Year | 2014 | 2015 | 2016 | 2017 | 2018 | 2019 |
|---|---|---|---|---|---|---|
| Projected population of women 15-49 | 23,198,829 | 24,084,767 | 24,949,201 | 25,832,132 | 26,725,476 | 27,620,498 |

b. It is very tricky to do random-effects models with survey weights. How the results would differ if you did not use weights. This could be placed in the supplements if indeed you did a random-effects model with sample weights.

- Thank you for the comment. As mentioned in line #140 and line #187 weights were used during the estimation of proportions and their respective confidence intervals. We did not consider sample weights when we fit the random-effects model.

8. The limitations should be part of the discussion as the reviewer points out below. Somewhere the last 3 paragraphs would be OK.

- Thank you. As most family planning research tends to include the limitations discussion near the end of the discussion section, we have inserted as the penultimate paragraph.
-

9. Thank you for the good plots. Just few things:

a. All of them should have the vertical y-axis start at zero. In ggplot2 there is something like expand_limits(y = 0) to sort that.

b. Make all of them to have the same maximum. It is easy to misunderstand the plots as they have different scales.

- Thank you for the helpful comments. In the revised version, the figure axis was updated accordingly.

**Reviewers' comments:**

**Abstract**

In methods section it is written “women aged 1549” but it should say “15 to 49”.

- Updated accordingly.

In results section what is meant by “the percentage of women that received high family planning counseling service…” Do you mean high quality FP services?

- Yes, thank you. This has been updated throughout.

I am not sure if I use the term “determinants” to characterize the factors that you found were associated with FP counseling quality. For example, I think media is a contextual factor that may be associated with experiencing better counseling quality, but I question whether it truly determines the level of counseling quality.

- We thank the reviewer for their suggestion, and have replaced “determinant factors” with “factors”.

Even though this may cause the authors to exceed the word limit for the abstract, I think the abstract would be strengthened if it briefly stated how the authors define FP counseling quality.

- Thank you. We have added this to the introduction section of the abstract.

**Background**

The new paragraph that points out that in a study on MII across 25 countries “residence, education, household wealth and method type were generally found to be associated with receiving higher quality care” … To be helpful, please specify residence where and what method types were associated with higher quality care.

- Thank you. We have clarified in the introduction section that urban residence was associated with receipt of higher quality care and included additional information on method type. The updated language on page 2 is copied below.

Despite substantial global efforts to improve conceptualization, measurement, and delivery of high-quality family planning services, studies consistently find that receipt of high-quality services is low. In a study exploring the levels and trends of the MII across 25 countries, urban residence, education, household wealth, and method type were generally found to be associated with receiving higher quality care [?]. In all countries combined, the median MII values show that the contraceptive information received by women varied by method type and was highest for implant users and lowest among women relying on sterilization.

In the same paragraph, when you say “individual level factors associated with receiving high quality care” it would be useful to clarify that you mean client-level (as opposed to individual provider-level).

- Thank you for this useful comment. We have rephrased the term “individual level” factors to “client-level” factors throughout the document.

**Materials and Methods**

Are you able to account for whether or not the survey participants were new or returning users? This matters since it is possible that returning users had already received the counseling information in recent FP visits at their nearby facility.

- Thank you for this question. The PMA surveys asked about the information that was received at the visit at which the client *first* received their particular contraceptive method. This has been clarified in the manuscript and the implications for the potential for recall bias has been included in the limitations section. We note that the PMA questions use the same question wording as the DHS when collecting information on the MII and thus, while this bias does exist, it is consistent with previous analyses.
-

To be helpful, specify what methods you define as long-acting and short-acting.

Thank you. This has been clarified in the methods section. Specifically, we define long-acting methods as female sterilization, implant and IUD and short-acting methods as the injectable, pill, emergency contraception, male condom, the Standard Days Method, and lactational amehorrhea method (LAM).

**Results**

“More than 70% of women in all years got received their contraceptive methods…” Check for grammar.

- Thank you, this has been updated.

Line 258 on page 8: you mention a decline in the proportion of survey participants that report good counseling services, but you say it is a decline in coverage. Elsewhere you call it percentage of women received high quality FP counseling. I question whether coverage is the best term for this. I think it is a measure of the prevalence. Please be consistent in how you refer to the measure.

- Thank you, we have updated throughout.

Line 262 on page 8 references Table?? – please correct this.

Updated.

Lines 255-267 page 9 – please check to see if you have used the correct tense.
Figure 2 label- “trends in getting high family planning counseling…” should it not say high quality family planning?

- Thank you. We have performed a thorough check of grammar throughout.

I understand the purpose of Table 4, but it is incredibly confusing. I suggest a table with a row for every year in the time series and columns for year, % change between successive years including the 95% CI in parentheses and cumulative % change between current and start year with 95% CI in parentheses.

- Thank you for the helpful suggestion. The table has been updated based on the following format.

Table X: Summary of observed percentage changes in high quality family planning counseling service across survey-years.

| **Reference/ base year** | **Survey year** | | | | |
|---|---|---|---|---|---|
| | **2015** | **2016** | **2017** | **2018** | **2019** |
| 2014 | 39.9(19.57,60.25) | 16.6(-3.03, 36.2) | 14.3(-8.52, 37.2) | 5.1(-16.1, 26.3) | -57.3(-67.11, -47.48) |
| 2015 | | -16.7(-28.10, -5.27) | -18.3(-34.7, -1.9) | -24(-40.1, 9.7) | -69.47(-76.5,-62.4) |
| 2016 | | | -2.0(22.02,18.17) | -9.84(-28.44,8.7) | -63.4(-71.9,-54.8) |
| 2017 | | | | -8.1(-21.84,4.69) | -62.65(-71.05,-54.25) |
| 2018 | | | | | -59.4(-68.56,-50.18) |

Lines 278-286- there seems to be some repetition here… please check and revise accordingly.

- Thank you, this has been updated.

Thanks for explaining the interpretation of Models A and B. After providing a thorough explanation of the interpretation for one of the explanatory variables, I do not think you need to repeat the same language over again for the other variables. But it is up to you.

-Thanks. This comment taken into account and we dropped the interpretation of some variables.

**Discussion**

It would be interesting if the authors could elaborate further on what might be the drivers of the decline in the outcome variable over the span of the time series.

- Thank you. We have added the following statement.

"Except in Addis Ababa, in all other regions, high-quality family planning counseling peaked in 2015 and declined since then in all other regions. The observed relatively better family planning counseling service in 2015 may be due to the fact the Ethiopian health sector transformation plan started in this year [31]. As compared with other regions, high quality counseling service was low in Amhara region and sharply declined since 2017 (Figure 2). Nationally, our finding on the trend analysis in the percentage of women receiving high quality family planning counseling service declined overtime. This aligns with Hrusa's finding using PMA data [12]."

I think it is good that in the discussion you mention that the association you identified between media exposure and the outcome is likely due to the fact that the former enhances knowledge and may prompt clients to ask more questions. In link 382 of page 15 you say this supports women in receiving high quality services, but I think a better way of phrasing it is that media exposure enables a more informative client-provider interaction because it's enhances client knowledge and empowers them to more inquisitive in the FP visit.

- Thank you, we have updated our language to better reflect the suggestion.

Can you establish whether women's reports of FP media exposure occurred before their reported FP visits in each survey? If not, then you should acknowledge that since women that received FP services may be more likely to seek out or recall FP-related media exposure (e.g., the visit peaked their interest, women that listen to educational media programs are more educated and therefore also more likely to use FP and ask questions during the visit). In other words, the direction of the association between media exposure and FP quality cannot be determined by this analysis, which limits the interpretation.
The abstract mentions that seeking FP counseling from pharmacies was associated with lower levels of FP counseling quality. This is an interesting finding and I think the authors should elaborate on this further in the discussion.

Thank you. In the discussion Section, we have added the following statement.
"Women who received care from pharmacies had significantly lower odds of receiveing high-quality family planning counseling than women who received care at either a health post or health center. In addition to differences in service modality and training of service providers, this likely reflects differences in characteristics of users and method choice; other studies have shown that women who use pharmacies tend to live in urban areas, be younger, and rely on short-term methods [38, 39]."

In the discussion, you mention that the odds of receiving high quality FP counseling are higher among women that obtain the service at public facilities compared to those that sought it at private facilities and refer to table 5 (lines 393-397 on page 16). But Table 5 does not include columns that refer to public vs. private facilities. Table 5 does give parameter estimates for health post, health center, pharmacy and other facility (ref hospital). This finding could be elaborated on in the discussion, with some clarification on the difference in the Ethiopian health system between health posts and health centers.

- Thank you. We clarify that the health posts, health centers, and hospitals included are all public health facilities, while pharmacies are all considered private facilities. We have provided additional information on page 12.

It is interesting that the outcomes vary so appreciably by region and if the authors have valuable insight on why this then please include that in the discussion.

-Added in the second paragraph of the discussion Section.

Lines 357-367 indicate that the analysis has implications regarding the need for youth friendly services, even though you found that age was not associated with FP counseling quality in the fully adjusted model. It seems that including parity and age in the same model washes out an

association between age and the outcome, which is not surprising. I do not think the findings give a useful interpretation related to provider biases and clients' age, and you should revise or remove this section from the discussion. Lines 419-430 are similarly confusing. Line 419 says age is not significantly associated with the outcome in the fully adjusted model, contrary to other studies and then you proceed to reflect on the crude OR in Model A. If after adjusting for parity, you find there is no age effect, then you might consider a different interpretation. For example, as women become more experienced with childbearing their knowledge and confidence during interactions with SRH providers increase and they are able to ask for and recall more information during their FP visits. I concede that women with little or no childbearing experience are also young, but I think you need to frame the discussion on age/parity and the outcome better and in a manner more aligned with Model B findings.

- Thanks for your fruitful comments. Accordingly updated.

**Limitations**

Unless it is a formatting requirement of the journal, I think the limitations sections should be included in the discussion section (personally, I think it is best at the beginning of the discussion). I do not think it is a good way to end the article.

- Thank you for the suggestion. We have included limitations in the discussion section, but have chosen to place them in the penultimate paragraph of the paper, as we found this was in keeping with the majority of family planning manuscripts included in our references.

I think the final sentence of the limitations should be expanded to state, specifically, that the analysis lacks really critical supply-side variables, such as availability of methods at facilities, availability of trained staff, job aids, information leaflets for clients, etc. Also, the analysis doesn't discuss whether survey participants were new or returning users, whether they had ever practiced FP in their lives, etc.

- Thank you. In the last line of the limitation paragraph, we included a statement which addresses the lack facility-level variables in the analysis and encourages further research on addressing this shortcoming.
-

**Conclusion and recommendation**

The conclusions right now seem rather generic and disconnected from specific lessons from the analysis. This is understandable but the authors need to reflect on what they can credibly conclude and give guidance on based on their findings and report this in a more focused manner.

The study seems to conclude that having more formal education, access to FP information from media and more experience with SRH care seeking (based on higher parity levels) is associated with recalling more FP counseling information. However, we also know from the analysis that counseling tends to be better at health posts and health centers compared to hospitals and is particularly poor at pharmacies, that LARC provision is association with better counseling, that some regions do appreciably better than others and that overall, counseling quality has declined over time.

-  Thank you.  We included more specific recommendations particularly highlighting the importance of providing additional training to pharmacists and related staff on contraceptive methods.

Though I am skeptical about this analysis because it does not include vital supply-side variable that affect counseling quality, I think the findings at least point in the direction of some supply- and demand-side actions that the MOH and partners should consider to address the problem of poor counseling quality.

A methodological recommendation to consider is incorporated more supply side measures (that PMA has made available from its extensive work conducting facilities assessments, I think) into future analysis on FP service quality.

Thank you.  We have added this recommendation in the limitations section.
